# Clinical Outcomes of Titanium Mesh for Alveolar Bone Augmentation: An Umbrella Review

**DOI:** 10.1002/cre2.70250

**Published:** 2025-12-10

**Authors:** Ethan Ng, Nikos Donos, Mohammad Adib Jaafar, John Rong Hao Tay, Nikos Mardas

**Affiliations:** ^1^ Faculty of Medicine and Dentistry Centre for Oral Clinical Research, Institute of Dentistry Queen Mary University of London London UK; ^2^ Department of Restorative Dentistry National Dental Centre Singapore Singapore Singapore; ^3^ Unit of Periodontics, Oral Health Programme, Ministry of Health Malaysia Putrajaya Malaysia; ^4^ Health Services Research and Population Health Programme, Duke‐NUS Medical School Singapore Singapore

**Keywords:** alveolar bone loss, alveolar ridge augmentation, bone grafting, bone regeneration, dental implants, titanium surgical mesh

## Abstract

**Objectives:**

To critically evaluate the evidence derived from systematic reviews for titanium mesh as a bone augmentation method for alveolar ridge deficiencies.

**Materials and Methods:**

Five databases were searched for systematic reviews describing any interventions to increase the dimensions of the alveolar ridge using titanium mesh. Included reviews had to report on the primary outcome of vertical and/or horizontal bone gain after augmentation with titanium mesh. The methodological quality of the systematic reviews was assessed with the AMSTAR 2 tool, and the certainty of evidence was evaluated with GRADE.

**Results:**

Eight systematic reviews were identified, including two with meta‐analysis. Of the 51 index studies included in the reviews, 20 were RCTs. The weighted mean reported vertical bone gain was 4.05 mm, and horizontal gain was 3.96 mm. However, overlap of primary studies across included reviews limits the precision of these estimates. When stratified by review quality, the single high‐quality review showed lower vertical and horizontal bone gain compared to the weighted mean. The average mesh exposure rate was 25%, although bone regeneration was usually still sufficient for implant placement.

**Conclusions:**

Titanium mesh can be effective for bone regeneration but carries a notable risk of site exposure. When choosing the technique for bone augmentation, clinicians should balance achieving optimal bone regeneration with minimal complications to enhance patient care and surgical outcomes.

## 1 Introduction

Adequate bone volume and quality is pivotal for successful implant placement and long‐term functionality (Monje et al. 2023; Cicciù et al. 2023; Donos et al. 2008). To address challenges arising from alveolar ridge defects after tooth extraction, alveolar bone augmentation procedures including guided bone regeneration (GBR) are commonly employed to restore bone volume and optimize conditions for implant placement (Jung et al. 2013; von Arx and Buser 2006; Ng et al. 2023). The concept of GBR is based on the principle that predictable bone regeneration can be achieved by using barrier membranes, allowing the creation of a secluded space for selective repopulation of the wound area with bone‐forming cells (Retzepi and Donos 2010).

Various types of membranes have been utilized in bone augmentation procedures to facilitate GBR (Mizraji et al. 2023; Calciolari et al. 2023; Donos et al. 2023). Resorbable membranes, composed of materials such as collagen or polylactic acid, offer advantages in terms of not requiring surgical re‐entry for removal, as well as ease of use. However, they are often incapable of maintaining the appropriate space needed to cover larger bone defects (Soldatos et al. 2017). Conversely, form‐stable non‐resorbable barrier devices such as titanium‐reinforced dense polytetrafluoroethylene (d‐PTFE) or titanium mesh (TM) provide a durable barrier that requires surgical removal once sufficient bone regeneration has occurred. These membranes are valued for their space‐maintaining capacity to maintain space over extended periods (Soldatos et al. 2017; Simion et al. 1994).

The use of TM as a technique for localized ridge augmentation developed in parallel to the use of the PTFE membrane (von Arx et al. 1996). TM functions as a rigid scaffold that maintains spatial dimensions for bone growth while preventing soft tissue encroachment (Xie et al. 2020). In comparison to resorbable barrier membranes, TM exhibits superior resistance to deformation and collapse, delivering enhanced mechanical strength and predictability in vertical augmentations (Xie et al. 2020; De Angelis et al. 2015; Rakhmatia et al. 2013).

Although TM has been extensively studied, outcomes vary, with some studies reporting successful bone regeneration, but others highlighting challenges associated with its use, such as its high rate of mesh exposure, infection, and bone resorption (Lizio et al. 2014). The discrepancies in results and methodologies suggest a need for a systematic evaluation of the evidence to clarify the role of TM in alveolar bone augmentation.

As there have been multiple systematic reviews published, an umbrella review is needed to synthesize the existing evidence comprehensively, to provide higher‐level insights to guide clinical decision‐making (Belbasis et al. 2022). Therefore, the objective of this umbrella review was to critically evaluate the use of TM in augmenting alveolar ridge deficiencies. This approach enables the appraisal and synthesis of the evidence, providing a comprehensive overview of current knowledge of the topic. Exemplary umbrella reviews are characterized by a clearly defined research question, predefined review selection criteria, a structured search strategy, systematic data extraction, critical appraisal of methodological quality of included systematic reviews, assessment of evidence strength, and a concise synthesis of key findings and their implications for clinical practice and research (Pollock et al. 2017; Smith et al. 2011). Nonetheless, challenges remain, including variability in the quality and reporting of included reviews, uncertainty over whether to reassess primary studies, and the absence of universally accepted methodological guidelines (Choi and Kang 2023).

## 2 Methods

### 2.1 Protocol Registration

A systematic approach was designed following the Cochrane structure for an overview of reviews (Pollock et al. 2024) and the protocol was registered in the PROSPERO database (registration number CRD42024604349).

### 2.2 Focused Question

The focused question for this umbrella review was “In patients with missing teeth who require bone augmentation to receive dental implants, what is the evidence for TM as a bone augmentation technique in terms of vertical and/or horizontal absolute bone gain?”

### 2.3 PICOS Framework

Based on the predefined focused question, the following components of PICOS were generated:

– Population: Patients with missing teeth who require vertical and/or horizontal alveolar bone augmentation with the intention to receive dental implants.
– Intervention: Any interventions to increase the dimensions of the alveolar ridge using any form of TM.
– Comparison: Various bone augmentation approaches using TM or other biomaterials.
– Outcomes: Absolute vertical and/or horizontal bone gain, implant survival, implant success, complications (e.g., mesh exposure, infection), implant placement feasibility, patient reported outcome measurements, the need for soft tissue augmentation procedures, aesthetic parameters, incidence of peri‐implant disease in implants placed in previously augmented with TM alveolar ridges, and histological findings.
– Study type: Systematic reviews with or without meta‐analysis.

### 2.4 Inclusion and Exclusion Criteria

The inclusion criteria were as follows:

– Systematic reviews on healthy individuals (ASA I–II), who underwent alveolar ridge augmentation (horizontal or/and vertical) using TM and were intended to receive dental implants.
– Included systematic reviews had to provide absolute bone gain in a vertical or horizontal dimension, or both.

The exclusion criteria were as follows:

– Conference abstracts, nonhuman studies, scoping reviews, and literature or narrative review articles.
– Studies on patients with severe systemic disease (ASA III–IV).
– Studies where TM was not the focus or where data were pooled with other interventions.
– Studies on ridge preservation in extraction sockets.

### 2.5 Search Strategy

To identify potential literature, a systematic search strategy was developed according to the PICOS framework. The initial strategy was built for PubMed using free text and MeSH keywords and then reformatted for other databases (Appendix 1). The following limits were placed: “English and humans.” A total of five databases were searched, including PubMed (Medline), Ovid (EMBASE), Cochrane Library, Scopus, and Web of Science. The initial electronic search was conducted on October 26, 2024 and updated on June 30, 2025. Hand searching of the reference lists of retrieved articles for further eligible reviews was also performed.

### 2.6 Selection Process

The search results were transferred to the citation manager EndNote 21 (Clarivate, Philadelphia, PA, USA) and duplicates were removed. A two‐stage selection process was carried out independently and in duplicate by two reviewers (E.N. and M.A.J.). Reviews were assessed based on their titles and abstracts first, and those that met the inclusion criteria were then screened for full‐text analysis. Any disagreement was resolved by discussion with a third reviewer (N.M.). The reviewers were not blinded to the author or institutions of the reviews undergoing screening.

### 2.7 Study Outcomes

The primary outcome was changes in vertical and/or horizontal dimensions of the alveolar ridge. The secondary outcomes were proportion of implant survival, implant success, complications (e.g., mesh exposure, infection), and implant placement feasibility; patient reported outcome measurements, incidence of peri‐implant disease, proportion requiring additional soft tissue augmentation procedures, any quantifiable aesthetic parameters, and histological findings.

### 2.8 Data Extraction and Management

Data were extracted independently by two reviewers (E.N. and M.A.J.) using a standardized data extraction form. The extracted data included study characteristics (author, year, journal of publication, and study design), study information (country, number and type of primary studies, PICO, details of search strategy, instrument of quality assessment, and risk of bias of primary studies), number of patients, intervention details (type of TM and grafting materials), and outcomes (absolute bone gain—vertical/horizontal, complications, implant success, implant survival, implant placement feasibility, need for further augmentation, histology, PROMs, incidence of peri‐implant disease, and histology). Authors of the included reviews were contacted directly for clarification, if required.

### 2.9 Quality Assessment of the Included Reviews

The AMSTAR 2 tool was used to assess the methodological quality of the systematic reviews (Shea et al. 2017). This is a validated tool that evaluates 16 domains to determine the quality of systematic reviews, (Lorenz et al. 2019) and is more widely used for assessing the methodological quality of systematic reviews, compared to the Risk of Bias in Systematic reviews tool (Bühn et al. 2017). Two review authors (E.N. and M.A.J.) independently assessed the risk of bias for all included reviews. Any disagreements between the review authors were resolved through discussion with a third reviewer (N.M.).

### 2.10 Data Synthesis

All included reviews were evaluated qualitatively, and the main findings were narratively summarized and reported in tables. The degree of study overlap was calculated using the corrected covered area method (Pieper et al. 2014). The certainty of evidence was assessed based on criteria within the Grading of Recommendations, Assessment, Development, and Evaluations (GRADE) adolopment framework (Pollock et al. 2014). Two review authors (E.N. and M.A.J.) independently rated the quality of the evidence, and any disagreements were resolved through discussion with a third reviewer (N.M.).

For each outcome (vertical bone gain, horizontal bone gain, and TM exposure), review‐level mean values were extracted and summarized descriptively. Exploratory dot plots were produced, where each point represents the reported mean from a systematic review. Both the simple (unweighted) mean and the sample‐size–weighted mean across reviews were calculated and displayed as vertical reference lines. All analyses were conducted in R (version 4.4.2; R Core Team, Vienna, Austria).

## 3 Results

### 3.1 Study Selection

**Figure 1:**
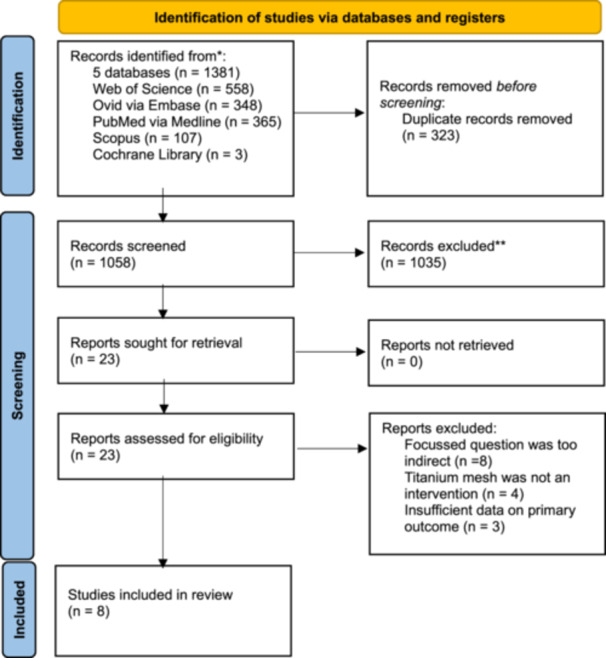
PRISMA flow diagram showing the study selection process.

A total of 1381 records were identified from five databases. No additional records were identified from the hand search; 1058 records were screened after removal of duplicates, 23 were assessed for full‐text, and finally 8 were included in this umbrella review (Figure 1). The list of excluded reviews with reasons may be found in Appendix 2.

### 3.2 Study Characteristics

Of the eight included systematic reviews (Rasia dal Polo et al. 2014; Briguglio et al. 2019; Aceves‐Argemí et al. 2021; Abu‐Mostafa et al. 2022; De Angelis et al. 2023; Mateo‐Sidrón Antón et al. 2024), two (Sabri et al. 2024; Lorusso et al. 2025) performed a meta‐analysis. All the reviews aimed to evaluate the use of TM during GBR, either as an intragroup comparison with different biomaterials or techniques to apply TM (Rasia dal Polo et al. 2014; Briguglio et al. 2019; Aceves‐Argemí et al. 2021), or an intergroup comparison with other membranes (Abu‐Mostafa et al. 2022; De Angelis et al. 2023; Mateo‐Sidrón Antón et al. 2024; Sabri et al. 2024; Lorusso et al. 2025). Bone gain in both the vertical and horizontal dimensions were evaluated by six reviews (Rasia dal Polo et al. 2014; Briguglio et al. 2019; Aceves‐Argemí et al. 2021; De Angelis et al. 2023; Mateo‐Sidrón Antón et al. 2024; Sabri et al. 2024), and two evaluated vertical changes alone (Abu‐Mostafa et al. 2022; Lorusso et al. 2025). Four reviews included case series (Rasia dal Polo et al. 2014; Briguglio et al. 2019; Aceves‐Argemí et al. 2021; Mateo‐Sidrón Antón et al. 2024) and four included randomized clinical trials or observational studies only (Abu‐Mostafa et al. 2022; De Angelis et al. 2023; Sabri et al. 2024; Lorusso et al. 2025). The characteristics of the included systematic reviews, including the risk of bias assessment of their included primary studies are summarized in Table 1.

**Table 1:** Characteristics of included systematic reviews.

First author, year, journal	Country	Type of review	Stated aim of review	PICO	Details of search strategy	Instrument of quality assessment and risk of bias of primary studies	Number and type of primary studies
Rasia dal Polo et al. (2014), Medicina Oral Patologia Oral Cirugia Bucal	Italy	Systematic review	Evaluate the reliability of titanium mesh as a device applied to horizontal and vertical bone augmentation procedures	Not stated	3 databases (PubMed/Medline, Embase, and Science Direct) Search terms‐ keywords English language only, studies from 1973–2013	Not stated	17 (10 case series, 7 observational studies)
Briguglio et al. (2019), International Journal of Dentistry	Italy	Systematic review	Evaluate the success rate of titanium mesh during bone regeneration techniques, survival and success rate of implants, and the predictability of this surgical technique	Not stated	1 database (PubMed) Search terms‐ keywords Articles written in English published from 1998–2018	Not stated	7 (6 observational studies, 1 case series)
Aceves‐Argemí et al. (2021), Coatings	Spain	Systematic review	Assess the use of titanium meshes during guided bone regeneration, the quantity of augmented bone, survival and success rates of implants, complications, and predictability of this surgical technique	P: Patients with partially or total edentulism candidates for GBR I: GBR through autologous and/or heterologous bone graft with titanium meshes C: Different grafting materials and techniques O: The success rate regarding quantity of augmented bone, complications, and predictability	4 databases (PubMed/Medline, Scielo, Scopus, and Cochrane Library) Search terms‐ keywords English or Spanish studies published between 2000 and 2021	Cochrane collaboration's risk of bias tools (RoB 2 and ROBINS‐I) 5/6 studies with low risk, 1/6 with high risk	21 (13 case series, 4 RCT, 2 observational studies, 2 NRCTs)
Abu‐Mostafa et al. (2022), The Journal of Contemporary Dental Practice	Saudi Arabia	Systematic review	Evaluate the effectiveness and treatment outcomes of the GBR procedure with titanium mesh for vertical bone augmentation	P: Completely or partially edentulous healthy patients with severe or moderate vertical atrophy I: Regeneration approaches with titanium mesh C: Various approaches using titanium mesh and bone graft materials O: Total bone gain, titanium mesh exposure, early titanium mesh removal, infection rate, implant failure, and bone graft extrusion	4 databases (PubMed/Medline, Scopus, Science Direct, and Cochrane Library) Search terms‐ keywords Articles published in English between 2011 and December 2021	Critical appraisal skills program (CASP) checklist 2/8 low risk, 4/8 some concerns, 2/8 high risk	8 (5 observational studies, 3 RCTs)
De Angelis et al. (2023), The Open Dentistry Journal	Italy	Systematic review	Evaluate randomized clinical trials focusing on the use of conventional or customized titanium mesh	P: Healthy patients with atrophic edentulous maxilla or mandible I: Bone augmentation with titanium meshes C: Customized devices and conventional titanium meshes O: Incidence of complications and total volume of regenerated bone	3 databases (PubMed/Medline, Cochrane Database of Systematic reviews, and Scopus) Search terms‐ keywords Articles published in English up to December 2022	Not specified	6 RCTs
Mateo‐Sidrón Antón et al. (2024), British Journal of Oral and Maxillofacial Surgery	Spain	Systematic review	Evaluate the success rate of the titanium mesh technique for the placement of dental implants	P: Systemically healthy, smokers and non‐smokers, edentulous and partially edentulous patients who had undergone previous surgical procedures or simultaneous procedures I: Titanium mesh for vertical and/or horizontal bone reconstruction for implant placement C: Other vertical and/or horizontal techniques for bone reconstruction O: Bone gain and resorption, histological and histomorphometric findings, survival and complication rates of implants and titanium meshes	3 databases (PubMed/Medline, Scopus, and Web of Science) Search terms‐MeSH terms Articles published in English from January 2000 to October 2023	Cochrane risk of bias (RoB 2) tool, Newcastle Ottawa Scale, and the Joanna Briggs Institute 2/6 low risk, 2/6 some concerns, 2/6 high risk	6 (3 observational studies, 2 RCTs, 1 case series)
Sabri et al. (2024), International Journal of Oral Implantology	United States	Systematic review and meta‐analysis	Explore the bone augmentation outcomes achieved when using various procedures and provide a comparison between titanium mesh and different barrier membranes	P: Healthy human adult patients requiring horizontal and/or vertical augmentation either as a staged approach or simultaneous to implant placement I: Titanium mesh C: Other resorbable or non‐resorbable membranes O: Vertical and/or horizontal bone gain, implant survival, marginal bone loss, and complications	5 databases (Embase, PubMed, Cochrane Library, ClinicalTrials.gov, and Scopus) Search terms‐ keywords and MeSH terms No restrictions on language, articles up to May 2023	Cochrane collaboration's risk of bias tools (RoB 2 and ROBINS‐I) 16/22 low risk, 6/22 some concerns	22 (18 RCTs, 4 observational studies)
Lorusso et al. (2025), Dentistry Journal	Italy	Systematic review and meta‐analysis	Investigate the clinical effectiveness of titanium meshes in bone regeneration procedures	P: Patients affected by severe bone ridge atrophy in need for a bone graft I: Titanium mesh regeneration procedure C: Bone regeneration with a resorbable membrane O: Vertical bone gain, mesh exposure	3 databases (PubMed, EMBASE, and Google Scholar) Search terms‐ keywords	Cochrane Collaboration's risk of bias tool (RoB)	4 RCTs

Abbreviations: GBR, guided bone regeneration; NRCT, non‐randomized clinical trial; RCT, randomized clinical trial.

### 3.3 Treatment Modalities

The descriptive findings of the included systematic reviews may be found in Table 2. The described interventions to use TM were conventional TM that was pre‐shaped on a model or shaped chairside (Rasia dal Polo et al. 2014; Briguglio et al. 2019), TM covered with a collagen membrane (Abu‐Mostafa et al. 2022; De Angelis et al. 2023; Mateo‐Sidrón Antón et al. 2024; Sabri et al. 2024; Lorusso et al. 2025), TM covered with platelet‐rich plasma (PRP) (Rasia dal Polo et al. 2014; Aceves‐Argemí et al. 2021; Mateo‐Sidrón Antón et al. 2024), or customized 3D‐printed TM (Aceves‐Argemí et al. 2021; Abu‐Mostafa et al. 2022; De Angelis et al. 2023; Mateo‐Sidrón Antón et al. 2024; Sabri et al. 2024; Lorusso et al. 2025). TM thickness was reported by three reviews, which ranged from 0.1 to 2 mm, with 0.2 mm being the most commonly used (Briguglio et al. 2019; Aceves‐Argemí et al. 2021; Abu‐Mostafa et al. 2022). The regenerative bone grafting materials used in the different primary studies consisted of autogenous bone alone, autogenous bone mixed with xenograft, autogenous bone mixed with allograft, xenograft with PRP, autogenous bone mixed with alloplast, allograft mixed with xenograft, recombinant human bone morphogenic protein‐2 (rhBMP‐2), allograft alone, alloplast alone, xenograft alone, and a blood clot alone. Two systematic reviews included a quantitative comparison to other types of membranes, and this was to collagen membranes (Sabri et al. 2024).

**Table 2:** Descriptive findings of included systematic reviews.

First author, year	No. of patients receiving titanium mesh	Categories of titanium mesh interventions (construct, type of graft, adjunctive membrane, surface characteristics)	Primary outcome‐ vertical changes (mm)	Secondary outcome‐ horizontal changes (mm)	Secondary outcome‐ mesh exposure (%)	Secondary outcome‐ premature mesh removal (%)	Secondary outcome‐ implant placement feasibility	Secondary outcome‐ implant survival (%) (follow‐up)	Secondary outcome‐ implant success (%) (Albrektsson et al. 1986) (follow‐up)	Secondary outcome‐ histology
Rasia dal Polo et al. (2014)	246 patients	Construct‐ manually shaped Types of graft‐ particulate autogenous bone, autogenous bone block with particulate autogenous bone, autogenous bone with xenograft, xenograft alone, blood clot alone Adjunctive membrane‐ PRP (Torres et al. 2010) Surface characteristics‐ not reported	The average reported bone gain was 4.9 with a range of 3.3–8.6	The average reported bone gain was 4.4 with a range of 3.8–5.7	16.1 ± 15.8	20.0	Implant placement was possible in all cases	90.0 (18–96 months)	100 (18–96 months)	NR
Briguglio et al. (2019)	154 patients	Construct‐manually shaped Types of graft‐autogenous bone alone, autogenous bone with xenograft, hydroxyapatite Adjunctive membrane‐ none Surface characteristics‐ mesh thickness 0.1–0.3 mm	Bone gain in the range of 2.6–8.1	Bone gain in the range of 3.8–4.3	34.8 ± 30.6	22.8	Mesh exposures resulted in less bone formation, but this did not affect implant placement feasibility	98.3 (36–96 months)	85.3 (36–96 months)	NR
Aceves‐Argemí et al. (2021)	382 patients	Construct‐ manually shaped and 3D printed (Sumida et al. 2015, Ciocca et al. 2018, Cucchi et al. 2020) Types of graft‐ autogenous bone, autogenous with xenograft, allograft, autogenous bone with allograft, autogenous bone with alloplast, xenograft, xenograft with PRP, alloplast Adjunctive membrane‐PRP (Torres et al. 2010) Surface characteristics‐ mesh thickness 0.1 to > 0.5 mm	The average reported bone gain was 4.1 with a range of 2.6–8.9	The average reported bone gain was 4.3 with a range of 3.1–8.6	28.6 ± 22.8	NR	Optimal management of membrane exposures permits sufficient bone regeneration without compromising the final treatment outcome	99.3 (12–96 months)	93.3 (12–96 months)	NR
Abu‐Mostafa et al. (2022)	122 patients	Construct‐ manually shaped and 3D printed (Ciocca et al. 2018, Chiapasco et al. 2021, Li et al. 2021) Types of graft‐ autogenous bone with xenograft, xenograft, xenograft block, autogenous bone with allograft Adjunctive membrane‐ collagen membrane (Cucchi et al. 2017, Chiapasco et al. 2021) Surface characteristics‐ Mesh thickness 0.2–2 mm	Bone gain in the range of 2.6–4.8	NR	25.7 ± 25.6	2.4–11.1 as reported by two studies	Mesh exposures did not typically affect subsequent implant placement	100 (6–88 months)	NR	NR
De Angelis et al. (2023)	128 patients	Construct‐ manually shaped and 3D printed (Cucchi et al. 2021) Types of graft‐ autogenous bone with xenograft, xenograft Adjunctive membrane‐ Collagen membrane (Cucchi et al. 2017, Cucchi et al. 2021, Cucchi et al. 2019), PRP (Torres et al. 2010) Surface characteristics‐ not reported	Bone gain in the range of 3.1–4.1	Bone gain of 3.9 based on one study	23.8 ± 9.0	NR	Implants were placed as planned, but two cases required additional bone grafting	NR	NR	NR
Mateo‐Sidrón Anton et al. (2024)	126 patients	Construct‐ manually shaped and 3D printed (Cucchi et al. 2021) Types of graft‐autogenous bone, xenograft with PRP, allograft, xenograft, autogenous bone with xenograft Adjunctive membrane‐ PRP (Torres et al. 2010), collagen membrane (Zhang et al. 2019, Cucchi et al. 2021) Surface characteristics‐ not reported	Bone gain in the range of 1.5–4.8	Bone gain in the range of 3.1–3.9	19.3 ± 12.9	NR	Bone gain was reduced in cases of exposure, but in nearly all cases this did not jeopardize insertion or survival of the implant	94–100(8–41 months)	NR	Histological analysis of regenerated sites showed mineralized newly‐formed bone, and some showed a connective tissue layer in the most coronal part
Sabri et al. (2024)	365 patients	Construct‐ manually shaped and 3D printed (Al Shaik et al. 2020, Cucchi et al. 2021, Sumida et al. 2015) Types of graft‐ autogenous bone with xenograft, autogenous bone with allograft, allograft with xenograft, rhBMP‐2, autogenous bone alone, particulate/block xenograft alone Adjunctive membrane‐ collagen membrane (Cucchi et al. 2023, Cucchi et al. 2019, Cucchi et al. 2021; Cucchi et al. 2017, Mounir et al. 2019, Cucchi et al. 2021) Surface characteristics‐ not reported	The average reported bone gain was 3.4 with a range of 1.4–5.7	The average reported bone gain was 3.3 with a range of 2.6–3.7	NR	NR	NR	NR	NR	No significant differences in mineralized and non‐mineralized tissue between d‐PTFE membranes resorbable membranes, and titanium mesh
Lorusso et al. (2025)	65 patients	Construct‐ manually shaped and 3D printed (Li et al. 2023, Cucchi et al. 2021) Types of graft‐ autogenous bone with xenograft Adjunctive membrane‐ collagen membrane (Cucchi et al. 2017, Cucchi et al. 2021) Surface characteristics‐ not reported	4.22 ± 1.68	NR	21.5	NR	NR	NR	NR	NR

Abbreviations: DBBM, demineralized bovine bone matrix; d‐PTFE, dense polytetrafluoroethylene; NR, not reported; PEEK, polyetheretherketone; PRP, platelet‐rich plasma; rhBMP‐2, recombinant human bone morphogenic protein‐2.

### 3.4 Assessment of Methodological Quality

An individual assessment of the quality of each systematic review was performed using the AMSTAR 2 tool (Appendix 3). Two reviews were found to be of critically low quality (Rasia dal Polo et al. 2014; Briguglio et al. 2019), five were of low quality (Aceves‐Argemí et al. 2021; Abu‐Mostafa et al. 2022; De Angelis et al. 2023; Mateo‐Sidrón Antón et al. 2024; Lorusso et al. 2025), and one was of high quality (Sabri et al. 2024).

The reasons for decreasing quality of the reviews included the absence of the PICO framework (Rasia dal Polo et al. 2014; Briguglio et al. 2019), not establishing a protocol a priori (Rasia dal Polo et al. 2014; Briguglio et al. 2019), the lack of a comprehensive literature search strategy (Briguglio et al. 2019), not providing a list of excluded primary studies justifying the exclusions (Rasia dal Polo et al. 2014; Briguglio et al. 2019; Aceves‐Argemí et al. 2021; Abu‐Mostafa et al. 2022; Mateo‐Sidrón Antón et al. 2024), not describing the included primary studies in adequate detail (Rasia dal Polo et al. 2014; Briguglio et al. 2019; De Angelis et al. 2023), not investigating publication bias (Lorusso et al. 2025), and not using a satisfactory technique to assess risk of bias in primary studies (Rasia dal Polo et al. 2014; Briguglio et al. 2019; De Angelis et al. 2023). Notably, only one review reported on the sources of funding for the included studies (Sabri et al. 2024).

**Figure 2:**
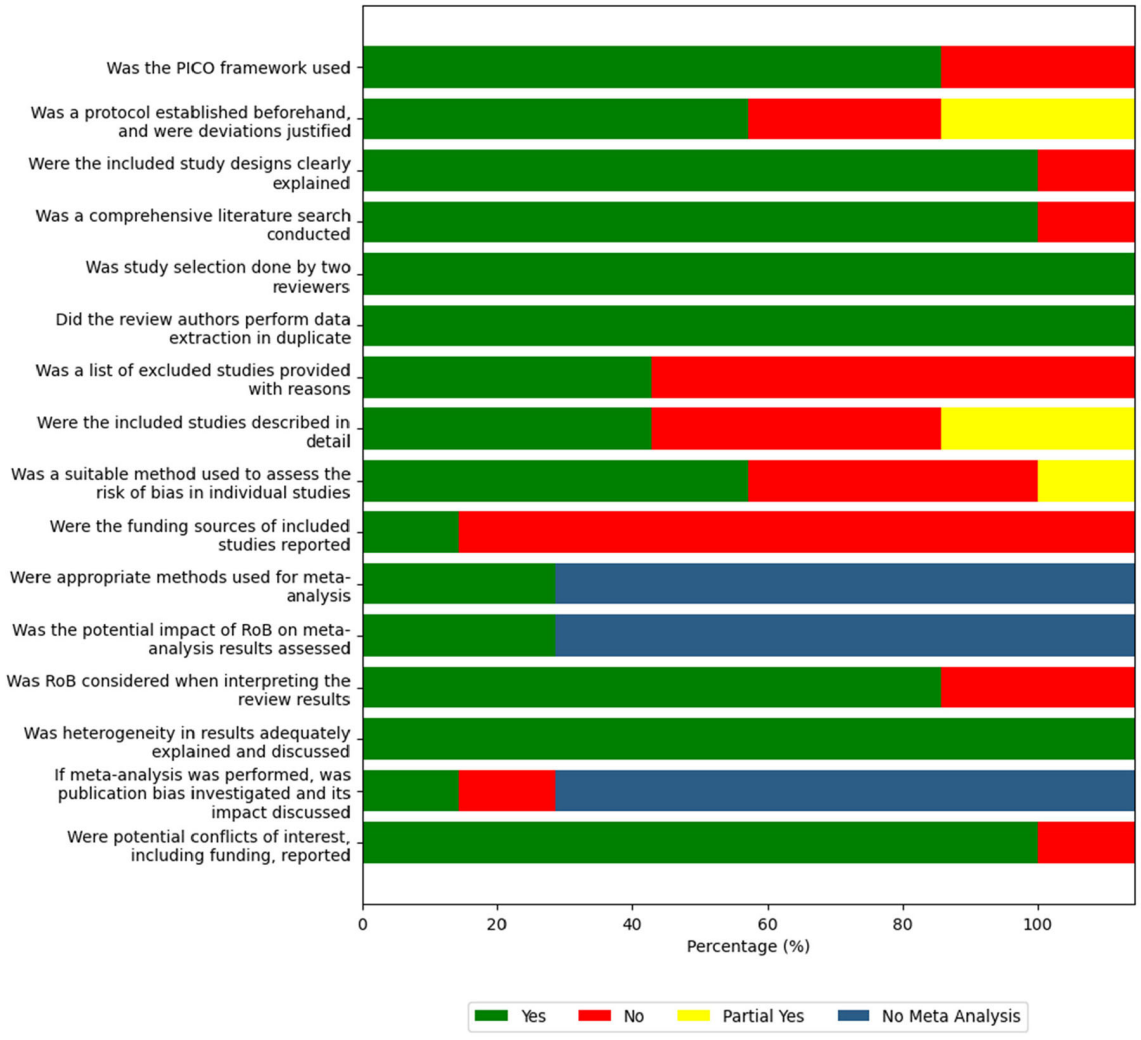
Summary results of AMSTAR 2 domains.

The compiled results of the included reviews for each AMSTAR 2 domain are summarized in Figure 2. Two studies (Aceves‐Argemí et al. 2021; Abu‐Mostafa et al. 2022) received a partial yes for item 8, as the information of included primary studies was not described with adequate detail. One study (Abu‐Mostafa et al. 2022) also received a partial yes for item 9 (use of a satisfactory technique for assessing the risk of bias) for using the critical appraisal skills program (CASP) checklist instead of the more established tools from the Cochrane collaboration.

### 3.5 Assessing Primary Study Overlap Within the Included Studies

**Figure 3:**
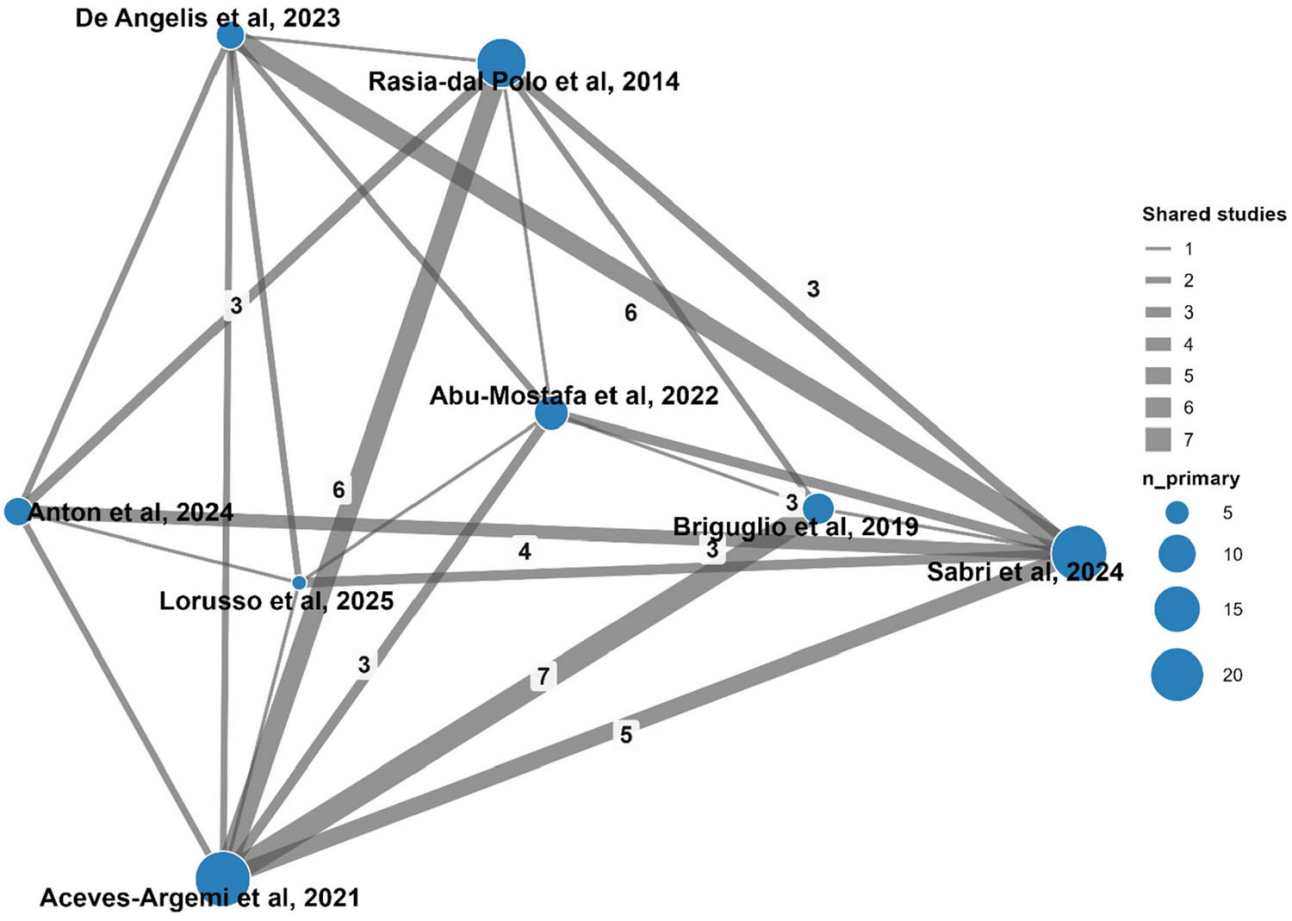
Node‐link graph of evidence overlaps among the included systematic reviews. The size of each node is proportional to the total number of primary studies included in the respective systematic review. The thickness of the lines indicates the number of shared primary studies between two linked systematic reviews. Numbers are displayed on the edges only when three or more primary studies are shared.

A citation matrix of the primary studies included in the systematic reviews was created to assess primary study overlap (Appendix 4). The number of included publications including double counting was 91, and the number of index publications was 51. This gave a corrected covered area of 11.2%, suggesting borderline high overlap. The network of included systematic reviews and overlapping primary studies is visually represented in Figure 3.

### 3.6 Assessing the Certainty of the Evidence

GRADE scores were not reported as part of the assessment in any of the systematic reviews. Therefore, GRADE assessments were conducted by the authors, using the information reported in the systematic reviews (Appendix 5). Among the included reviews, one was of very low certainty (Briguglio et al. 2019), four were low (Rasia dal Polo et al. 2014; Abu‐Mostafa et al. 2022; De Angelis et al. 2023; Mateo‐Sidrón Antón et al. 2024), and three had a moderate level of certainty (Aceves‐Argemí et al. 2021; Sabri et al. 2024; Lorusso et al. 2025).

### 3.7 Primary and Secondary Outcomes

**Figure 4:**
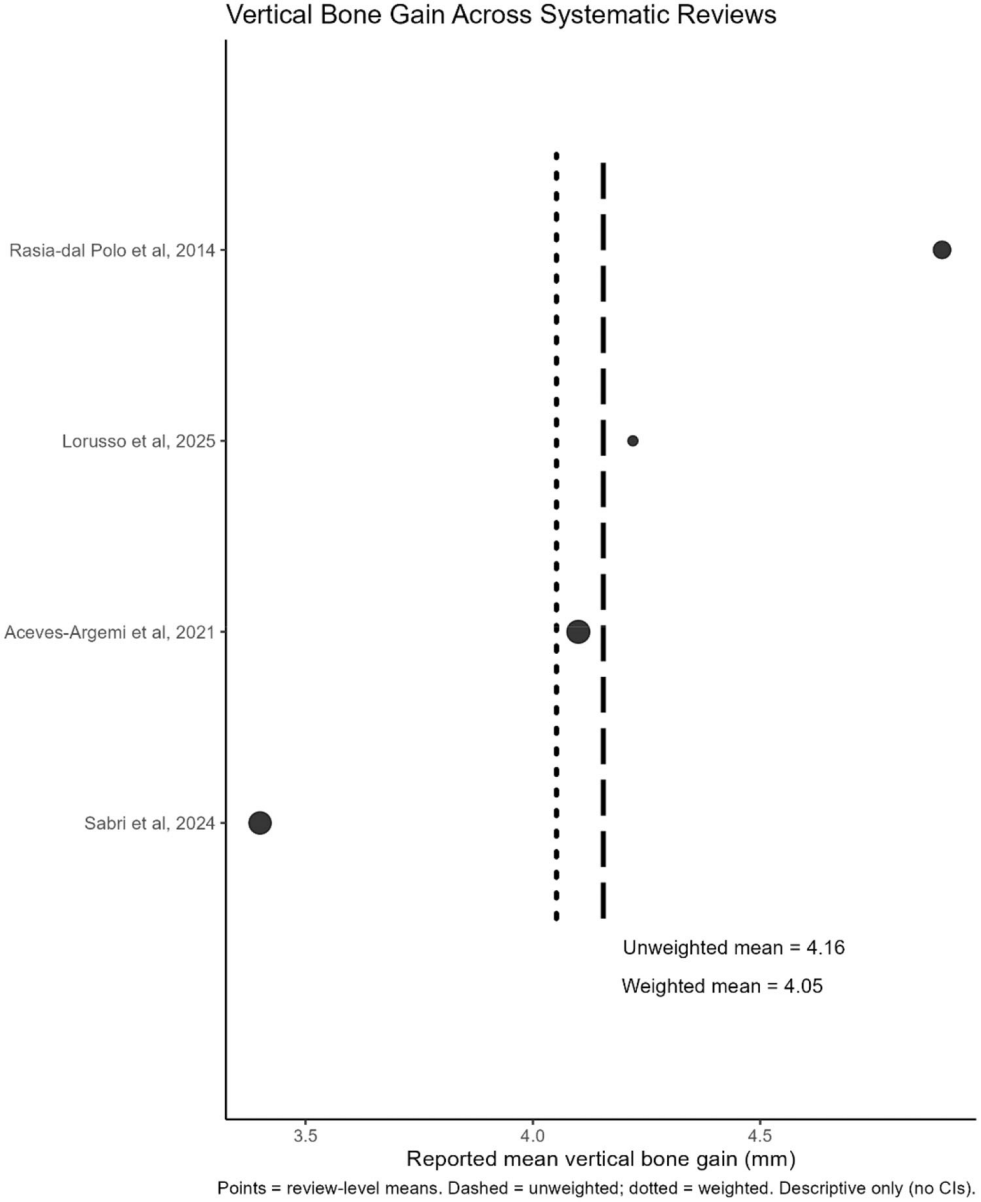
Vertical bone gain across systematic reviews (mm).

**Figure 5:**
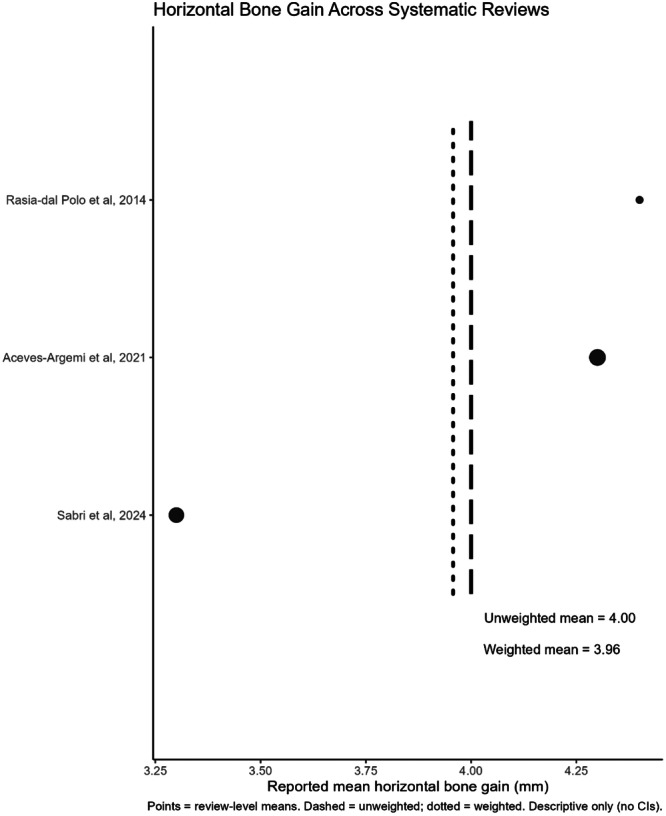
Horizontal bone gain across systematic reviews (mm).

The primary and secondary outcome data are summarized in Table 3. All eight of the included reviews reported on the primary outcome of vertical bone gain. Four studies (Rasia dal Polo et al. 2014; Aceves‐Argemí et al. 2021; Sabri et al. 2024; Lorusso et al. 2025) indicated a weighted mean vertical bone gain of 4.05 mm (Figure 4). Of the six reviews reporting on the primary outcome of horizontal bone gain. Three studies (Rasia dal Polo et al. 2014; Aceves‐Argemí et al. 2021; Sabri et al. 2024) indicated a weighted mean horizontal bone gain of 3.96 mm (Figure 5).

**Table 3:** Summary of findings table.

**1. Primary outcome‐ Vertical changes (mm)**
**The body of evidence suggests** that titanium mesh results in gains in vertical dimensions, with high variability. The unweighted mean bone gain as reported by four studies was 4.2, and the mean weighted mean bone gain was 4.1 mm. **Supporting literature** 2 systematic reviews and 2 meta‐analyses. **Quality assessment** 1 critically low, 2 low quality systematic reviews, and 1 high quality meta‐analysis. **Grade of evidence** Moderate.
**2. Primary outcome‐ Horizontal changes (mm)**
**The body of evidence suggests** that titanium mesh results in gains in horizontal dimensions, with high variability. The unweighted and weighted mean bone gain as reported by three studies was 4.0. **Supporting literature** 2 systematic reviews and 1 meta‐analysis. **Quality assessment** 1 critically low, 1 low quality systematic review, and 1 high quality meta‐analysis. **Grade of evidence** Moderate.
**3. Secondary outcome‐ Mesh exposure (%)**
**The body of evidence suggests** that titanium mesh can result in an average mesh exposure of 16%–35%. The unweighted mean titanium mesh exposure rate based on seven studies was 24.3% and the weighted mean was 24.7%. Mesh exposure can occur early (within 4 weeks) or late (> 4 weeks) but does not always necessitate removal. **Supporting literature** 6 systematic reviews and 1 meta‐analyses. **Quality assessment** 2 critically low and 5 low quality systematic reviews. **Grade of evidence** Low‐moderate.
**4. Secondary outcome‐ Implant placement feasibility**
**The body of evidence suggests** that despite mesh exposures, bone regeneration was usually still sufficient to facilitate implant placement. **Supporting literature** 6 systematic reviews. **Quality assessment** 2 critically low and 4 low quality systemic reviews. **Grade of evidence** Low.
**5. Secondary outcome‐ Implant survival (%)**
**The body of evidence suggests** that placing implants in sites grafted using titanium mesh as a barrier result in an implant survival rate of 90%–100% (6–96 months). **Supporting literature** 5 systematic reviews. **Quality assessment** 2 critically low and 3 low quality systemic reviews. **Grade of evidence** Low.
**6. Secondary outcome‐ Implant success (%)**
**The body of evidence suggests** that placing implants in sites grafted using titanium mesh as a barrier result in an implant success rate of 85%–100% (12–96 months). **Supporting literature** 3 systematic reviews. **Quality assessment** 2 critically low and 1 low quality systemic review. **Grade of evidence** Low.
**7. Secondary outcome‐ Histology**
**The body of evidence suggests** no significant differences in mineralized and non‐mineralized tissue between titanium mesh and d‐PTFE membranes or resorbable membranes. **Supporting literature** 2 systematic reviews. **Quality assessment** 1 low quality systemic review and 1 high quality meta‐analysis. **Grade of evidence** Low‐moderate.

**Figure 6:**
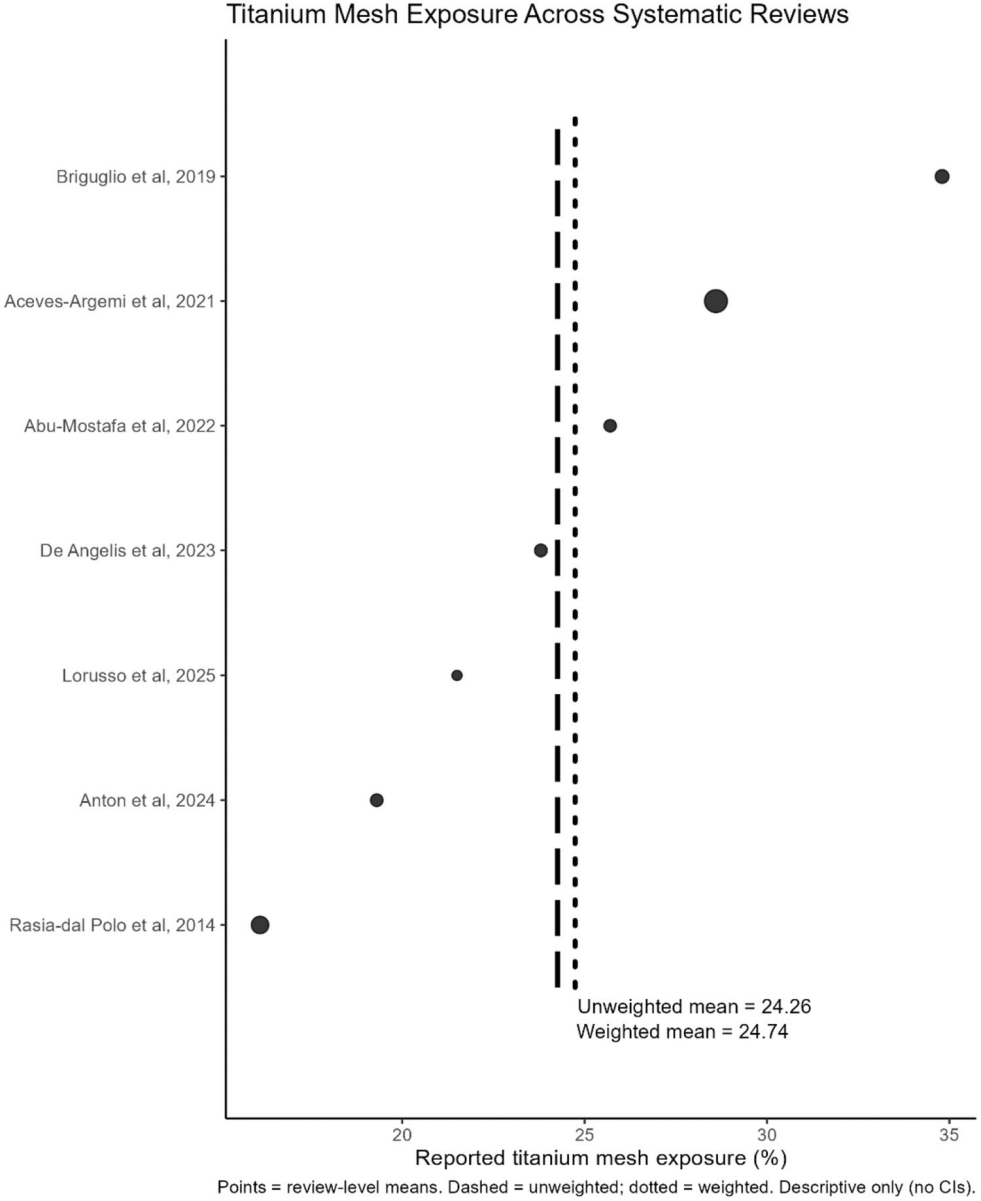
Titanium mesh exposure across systematic reviews.

The use of TM was associated with an average mesh exposure rate in the range of 16%–35%, with high observed heterogeneity. Seven studies (Rasia dal Polo et al. 2014; Briguglio et al. 2019; Aceves‐Argemí et al. 2021; Abu‐Mostafa et al. 2022; De Angelis 2023; Mateo‐Sidrón Antón et al. 2024; Lorusso et al. 2025) indicated a weighted exposure rate of 24.7% (Figure 6). Mesh exposures could occur early (within 4 weeks) or late (> 4 weeks) (Abu‐Mostafa et al. 2022; De Angelis et al. 2023; Sabri et al. 2024). Premature TM removal in patients usually occurred between 3 and 7 months, and was reported at 20% based on five primary studies (Rasia dal Polo et al. 2014), 23% based on three primary studies (Briguglio et al. 2019), and 2.4%–11% based on two primary studies (Abu‐Mostafa et al. 2022).

Implant placement feasibility was possible in most cases, even when mesh exposures had occurred (Rasia dal Polo et al. 2014; Briguglio et al. 2019; Aceves‐Argemí et al. 2021; Abu‐Mostafa et al. 2022; De Angelis et al. 2023; Mateo‐Sidrón Antón et al. 2024). However, one systematic review reported a primary study where additional bone grafting was required for two cases (De Angelis et al. 2023). Implant placement in sites grafted using the TM technique resulted in an implant survival rate of 90%–100% based on five reviews (Rasia dal Polo et al. 2014; Briguglio et al. 2019; Aceves‐Argemí et al. 2021; Abu‐Mostafa et al. 2022; Mateo‐Sidrón Antón et al. 2024). The shortest follow‐up time after implant placement was 8 months, and the longest was 96 months. Three reviews reported an implant success rate of 85%–100% (Rasia dal Polo et al. 2014; Briguglio et al. 2019; Aceves‐Argemí et al. 2021), using Albrektsson's criteria (Albrektsson et al. 1986). The shortest follow‐up time was 12 months, and the longest was 96 months after implant placement. Two reviews included histological analysis of the tissues at the regenerated sites (Mateo‐Sidrón Antón et al. 2024; Sabri et al. 2024). While some primary studies reported a connective tissue layer in the most coronal aspect, there were no significant differences in mineralized and non‐mineralized tissue compared to d‐PTFE or resorbable membranes. There were no data available on patient reported outcome measurements, incidence of peri‐implant disease, the need for soft tissue augmentation procedures, and aesthetic parameters.

## 4 Discussion

### 4.1 Summary of the Main Results

This umbrella review sought to present and describe the current body of systematic review evidence on the use of TM in GBR. Based on evidence from eight systematic reviews, including two with meta‐analysis, TM as a technique demonstrates efficacy in both vertical and horizontal bone augmentation. However, the technique is associated with a significant mesh exposure rate, although implant placement was still possible despite reduced bone gain. The substantial heterogeneity observed in the bone regeneration outcomes and complication rate underscores the technical sensitivity of this procedure, which demands advanced surgical skill and experience.

### 4.2 Agreements and Disagreements With Other Studies or Reviews

The weighted mean vertical bone gain reported by four studies was 4.05 mm, and horizontal bone gain was 3.96 mm based on three studies. These findings are comparable to the findings from other meta‐analyses where other augmentation techniques besides TM have been used. Specifically, pooled weighted mean gain for different augmentation techniques have been reported as 4.2 mm for vertical bone regeneration (Urban et al. 2019) and 3.5 mm for horizontal bone regeneration (Naenni et al. 2019).

The weighted mean exposure rate of TM was 24.7% based on seven studies. The observation that up to 1 in 4 cases involving TM may result in mesh exposure is consistent with the findings of other systematic reviews which have reported an average mesh exposure rate of 33% (Cunha et al. 2022), 29% (Poli et al. 2022), and at least 20% (Gu et al. 2022). This is a clinically important finding, as a significant negative correlation has been reported between the amount of bone gain and the area of exposed TM, with approximately 30% less regenerated bone expected in such cases (Lizio et al. 2014). Similarly, a recent systematic review with meta‐analysis reported that membrane exposures were associated with a 35% decrease in vertical bone gain (Tay et al. 2022). While a cause‐and‐effect relationship between membrane exposure and bone loss may be inferred, the timing of the exposure critically influences bone gain (Watzinger et al. 2000). Interestingly, based on six reviews (Rasia dal Polo et al. 2014; Briguglio et al. 2019; Aceves‐Argemí et al. 2021; Abu‐Mostafa et al. 2022; De Angelis et al. 2023; Mateo‐Sidrón Antón et al. 2024), although a mesh exposure reduced the overall amount of bone formation, its occurrence did not jeopardize implant placement in most cases even if simultaneous GBR was required.

Based on two reviews included in this study, premature removal of TM due to complications is 20%–23%, closely mirroring the rate of 21% in another systematic review (Cunha et al. 2022). This suggests that 1 in 5 cases involving TM require premature removal before the planned re‐entry date, often due to complications arising from exposure with concurrent infection. An early exposure resulting in premature removal within the first 4 weeks postoperation frequently resulted in either complete treatment failure or insufficient bone regeneration; in contrast, late mesh exposures unaccompanied by infection may not necessitate removal (Cucchi et al. 2017; Corinaldesi et al. 2009). In the absence of infection, titanium meshes may be able to tolerate a certain degree of exposure as their porous structure allows for spontaneous healing of the underlying mucosa and the grafted bone can remain sufficiently stabilized by newly formed bone (Briguglio et al. 2019; Aceves‐Argemí et al. 2021; Abu‐Mostafa et al. 2022). In these situations, maintaining cleanliness of the grafted area, typically using chlorhexidine, and oral hygiene instructions with regular follow‐up is the recommended management approach (Cunha et al. 2022).

Four of the included systematic reviews (Aceves‐Argemí et al. 2021; Abu‐Mostafa et al. 2022; De Angelis et al. 2023; Sabri et al. 2024) included primary studies which utilized digitally customized titanium meshes. While this could simplify the procedure and reduce surgical time compared to using a conventional TM, the evidence regarding the advantages of customized meshes in terms of reducing healing complications was inconclusive. Indeed, the existing evidence for the superiority of a customized mesh regarding mesh exposure is also equivocal. While one other review found no significant difference in exposure rate between conventional and customized titanium meshes (Gu et al. 2022), another reported a 20% reduction in exposure rates (Zhou et al. 2022). Nevertheless, high exposure rates of 31% (Zhou et al. 2022) and 25% (Hartmann and Seiler 2020) have been documented even with customized meshes. These exposure rates are comparable to those observed with conventional meshes in this umbrella review, suggesting that certain clinical challenges related to soft tissue management cannot be fully mitigating using 3D printing and mesh customization alone.

### 4.3 Assessment of Included Reviews

The AMSTAR 2 quality assessment revealed that most of the included systematic reviews exhibited serious methodological limitations, resulting in “critically low” or “low” quality ratings. Common shortcomings of earlier systematic reviews included the lack of justification for excluded studies, insufficient description of included studies, inadequate methods for assessing risk of bias, and the omission of funding source disclosures for primary studies. In contrast, systematic reviews published within the past 5 years were more likely to demonstrate improved methodological rigor, including prospective protocol registration and adherence to PRISMA guidelines.

While AMSTAR 2 is a validated tool for evaluating the quality of systematic reviews, it does not capture certain structural and methodological nuances. For instance, the tool accepts any reported search strategy as adequate, without evaluating whether it includes a sufficient combination of both MeSH terms and free‐text keywords. Additionally, the type of studies selected for inclusion significantly impacts the quality and interpretability of a systematic review. In the present umbrella review, several systematic reviews combined data from heterogeneous study designs including retrospective case series, case reports, observational studies, and RCTS, leading to increased heterogeneity and limited potential for meta‐analysis. Prioritizing higher‐quality study designs and applying more uniform inclusion criteria would enhance the reliability and generalizability of the findings (Sabri et al. 2024).

### 4.4 Implications for Clinical Practice

Customized titanium meshes have been gaining popularity due to their ability to better conform to a patient's individual anatomy, potentially reducing the risk of mesh exposure (Scribante et al. 2023). However, while digital workflows may improve the precision of the mesh fit and reduce surgical time, they do not appear to mitigate the risk of mesh exposure. Indeed, the observed complication rates associated with TM technique are higher than those reported for other GBR approaches‐ 16% (Tay et al. 2020) and 23% (Thoma et al. 2019) for horizontal augmentation, and 11% (Tay et al. 2022) and 17% (Urban et al. 2019) for vertical augmentation.

Clinical experience plays a pivotal role in managing factors that influence mesh exposure, such as soft tissue management, vestibular depth, flap thickness, the extent of flap release, and advancement and stabilization of the site with appropriate suturing techniques. This aligns with findings from a recently conducted network meta‐analysis on various surgical techniques for managing vertical ridge defects, which identified operator experience (> 200 procedures) as a critical factor influencing complication rates (Alotaibi et al. 2023). Techniques yielding greater bone gain often correlate with higher complication rates (Urban et al. 2019). Therefore, resorbable membranes may be suitable when less extensive augmentation is required, whereas d‐PTFE membranes demonstrate superior performance as space‐maintaining barriers in scenarios requiring greater stability, as highlighted in the same network meta‐analysis (Alotaibi et al. 2023).

### 4.5 Implications for Future Research

Future clinical research should aim to generate more comprehensive evidence, particularly by incorporating patient‐reported outcome measures (PROMS) (Tonetti et al. 2023). PROMs offer valuable insights that support shared decision‐making and are gaining importance alongside biological and clinical measures (Ng et al. 2021). To date, only one study has explored this aspect, reporting favorable outcomes when comparing a customized reinforced PTFE mesh to a CAD/CAM TM (Cucchi et al. 2025). Standardizing the reporting of outcomes across clinical studies, such as healing complications and changes in bone dimensions, would also enhance the ability to assess overall effectiveness. Additionally, more detailed reporting on the characteristics of regenerated bone including implant stability at placement, correlation between timing of mesh exposure and premature removal, the need for additional hard or soft tissue grafting, and long‐term implant success and survival is currently lacking and should be prioritized in future studies.

### 4.6 Strengths and Limitations

By conducting a comprehensive umbrella review, we provided a broad overview of the effectiveness of TM for bone augmentation across a wide range of clinically relevant primary and secondary outcomes. A notable strength of this study is the rigorous methodology used to identify relevant literature, which included a systematic search across five databases; and to appraise the literature, which included AMSTAR 2, GRADE, and the calculation of overlap.

Crucially, this is the first review to assess the quality of individual systematic reviews and the certainty of evidence within the oral bone reconstruction domain. Despite systematic reviews being positioned at the top of the evidence hierarchy, the AMSTAR 2 assessment revealed that many of the included reviews exhibited significant methodological limitations. These shortcomings undermine the reliability and strength of the evidence currently available to guide clinical decision‐making.

This umbrella review is not without limitations. The validity of an umbrella review is inherently tied to the scope and quality of the primary studies it synthesizes. Methodological flaws and biases in the original research may be compounded in umbrella reviews, making them more difficult to address at a secondary level. Furthermore, heterogeneity in outcome reporting limited the extraction of comparable data. In many instances, the anatomical site of intervention could not be clearly determined, and information regarding defect dimensions or severity was inconsistently reported. These limitations preclude reliable indirect comparisons between specific TM interventions or between alternative GBR approaches. This highlights the need for future systematic reviews to have greater methodological standardization and transparent reporting to enable more robust and interpretable evidence synthesis in umbrella reviews.

The inclusion of overlapping reviews introduces another potential limitation, as data from the same primary study may be included multiple times. However, given the design of this study, the inclusion of all the relevant systematic reviews regardless of overlap was deemed appropriate (Pollock et al. 2024). Conducting GRADE assessments at the overview level using data derived from systematic reviews is particularly challenging, as key information required to evaluate evidence quality is often missing or inadequately reported (Pollock et al. 2016). Therefore, adaptations to the GRADE tool, as implemented in this study, may be necessary for its application in a review of systematic reviews (Pollock et al. 2014).

## 5 Conclusions

Despite variability in methodological quality, the systematic reviews included in this umbrella review consistently support the efficacy of TM for bone augmentation, though this is tempered by a notably high rate of mesh exposure. The considerable heterogeneity in reported outcomes highlights the technical sensitivity of the procedure, reinforcing the need for careful case selection and a high level of surgical expertise.

## Author Contributions

Ethan Ng contributed to conception, development of the protocol, data acquisition, visualization, and interpretation; and drafted and critically revised the manuscript. Nikos Donos conceived the idea, contributed to development of the protocol, and interpretation; and critically revised the manuscript. Mohammad Adib Jaafar contributed to data acquisition, visualization, and interpretation; and critically revised the manuscript. John Rong Hao Tay contributed to analysis, visualization, and interpretation; and critically revised the manuscript. Nikos Mardas contributed to conception, development of the protocol, and interpretation; and critically revised the manuscript. All authors gave their final approval and agreed to be accountable for all aspects of work.

## Conflicts of Interest

The authors declare no conflicts of interest.

## Supporting information


**Appendix 1:** Systematic search strategy built for PubMed (Medline) using a combination of MesH keywords and text words.

**Appendix 2:** Table of excluded studies with reasons.

**Appendix 3:** AMSTAR 2 assessment tool.

**Appendix 4:** Citation matrix of primary studies included in the systematic reviews (total *n* = 51).

**Appendix 5:** Grading the evidence.

## Data Availability

The data that supports the findings of this study have been included in the manuscript and the appendices.
