# Supplementary material for: Clinical Outcomes of Titanium Mesh for Alveolar Bone Augmentation: An Umbrella Review
Source: Clin Exp Dent Res. 2025 Dec 10;11(6):e70250. doi: 10.1002/cre2.70250 (PMC12690612; doi:10.1002/cre2.70250)
Supplement: Supplementary file 1 — Appendix 1: Systematic search strategy built for PubMed (Medline) using a combination of MesH keywords and text words. [file CRE2-11-e70250-s003.docx]

**Appendix 1. Systematic search strategy built for PubMed (Medline) using a combination of MesH keywords and text words**

|  | MeSH | Text word |
| --- | --- | --- |
| Population | “dental implants” OR “alveolar bone loss” OR “alveolar bone atrophy” OR “alveolar resorption” | “deficient ridge” OR “atrophic ridge” OR “bone dehiscence” |
| Intervention/comparison | “surgical mesh” OR “alveolar ridge augmentation” OR “bone regeneration” OR “bone grafting” | “Titanium mesh” OR “occlusive titanium barrier” OR “titanium foil” OR “titanium membrane” OR “guided bone regeneration” OR “bone augmentation” OR “horizontal bone augmentation” OR “lateral bone augmentation” OR “vertical bone augmentation” |
| Filters | “systematic review” OR “meta-analysis” |  |
