# Supplementary material for: Clinical Outcomes of Titanium Mesh for Alveolar Bone Augmentation: An Umbrella Review
Source: Clin Exp Dent Res. 2025 Dec 10;11(6):e70250. doi: 10.1002/cre2.70250 (PMC12690612; doi:10.1002/cre2.70250)
Supplement: Supplementary file 2 — Appendix 2: Table of excluded studies with reasons. [file CRE2-11-e70250-s002.docx]

Appendix 2. Table of excluded studies with reasons

| Excluded study | Reasons |
| --- | --- |
| Carvalho et al 2019 | Focussed question was too indirect |
| De Groot et al 2018 | Titanium mesh was not an intervention |
| Elnayef et al 2018 | Focussed question was too indirect |
| Elnayef et al 2017 | Focussed question was too indirect |
| Hameed et al 2019 | Titanium mesh was not an intervention |
| Naenni et al 2018 | Titanium mesh was not an intervention |
| Toti et al 2017 | Focussed question was too indirect |
| Ricci et al 2013 | Insufficient data on primary outcome |
| Smeets et al 2022 | Titanium mesh was not an intervention |
| Urban et al 2019 | Focussed question was too indirect |
| Zhang et al 2022 | Focussed question was too indirect |
| Alotaibi et al 2023 | Focussed question was too indirect |
| Roca-Millan et al 2020 | Insufficient data on primary outcome |
| Alotaibi et al 2025 | Focussed question was too indirect |
| H. Tang and Y. Zhang 2025 | Insufficient data on primary outcome |
