# Supplementary material for: Clinical Outcomes of Titanium Mesh for Alveolar Bone Augmentation: An Umbrella Review
Source: Clin Exp Dent Res. 2025 Dec 10;11(6):e70250. doi: 10.1002/cre2.70250 (PMC12690612; doi:10.1002/cre2.70250)
Supplement: Supplementary file 4 — Appendix 4: Citation matrix of primary studies included in the systematic reviews (total n = 51). [file CRE2-11-e70250-s004.docx]

Appendix 4. Citation matrix of primary studies included in the systematic reviews (total n = 51)

|  | Rasia-dal Polo et al, 2014 | Briguglio et al, 2019 | Aceves-Argemi et al, 2021 | Abu-Mostafa et al, 2022 | De Angelis et al, 2023 | Anton et al, 2024 | Sabri et al, 2024 | Lorusso et al, 2025 |
| --- | --- | --- | --- | --- | --- | --- | --- | --- |
| Malchiodi et al, 1998 | X |  |  |  |  |  |  |  |
| Leghissa et al, 1999 | X |  |  |  |  |  |  |  |
| Von Arx et al 1996 | X |  |  |  |  |  |  |  |
| Von Arx et al 1998 | X |  |  |  |  |  |  |  |
| Von Arx et al, 1999 | X |  |  |  |  |  |  |  |
| Assenza et al 2001 | X |  |  |  |  |  |  |  |
| Artzi et al, 2003 | X |  |  |  |  |  |  |  |
| Roccuzzo et al, 2004 | X |  |  |  |  |  |  |  |
| Proussaefs et al, 2006 | X | X | X |  |  |  |  |  |
| Roccuzzo et al, 2007 | X |  |  |  |  | X | X |  |
| Pieri et al, 2008 | X |  | X |  |  |  |  |  |
| Corinaldesi et al, 2009 | X | X | X |  |  |  |  |  |
| Torres et al, 2010 | X |  | X |  | X | X | X |  |
| Misch et al 2011 | X |  |  |  |  |  |  |  |
| Degidi et al, 2003 | X |  | X |  |  |  |  |  |
| Her et al, 2012 | X |  | X | X |  |  |  |  |
| Maiorana et al, 2021 | X |  |  |  |  | X | X |  |
| Corinaldesi et al, 2007 |  | X | X |  |  |  | X |  |
| Lizio et al, 2014 |  | X | X |  |  |  |  |  |
| Poli et al, 2014 |  | X | X | X |  |  |  |  |
| Miyamoto et al, 2011 |  | X | X |  |  |  |  |  |
| Uehara et al, 2015 |  | X | X |  |  |  |  |  |
| Bassi et al, 2016 |  |  | X |  |  |  |  |  |
| Pinho et al, 2006 |  |  | X |  |  |  |  |  |
| Sumida et al, 2014 |  |  | X |  |  |  | X |  |
| Zita et al, 2016 |  |  | X |  |  |  |  |  |
| Ciocca et al, 2018 |  |  | X | X |  |  |  |  |
| Cucchi et al, 2019 |  |  | X |  | X |  | X |  |
| Zhang et al, 2019 |  |  | X |  |  | X |  |  |
| Atef et al, 2020 |  |  | X |  |  |  | X | X |
| Malik et al, 2020 |  |  | X |  |  |  |  |  |
| Cucchi et al, 2020 |  |  | X |  |  |  |  |  |
| Mounir et al, 2017 |  |  |  | X |  |  | X |  |
| Cucchi et al, 2017 |  |  |  | X | X |  | X | X |
| Mounir et al, 2019 |  |  |  | X | X |  | X |  |
| Chiapasco et al, 2021 |  |  |  | X |  |  |  |  |
| Li et al, 2021 |  |  |  | X |  |  |  |  |
| Cucchi et al, 2021a |  |  |  |  | X | X | X | X |
| Cucchi et al, 2021b |  |  |  |  | X |  | X |  |
| Konstantinidis et al, 2015 |  |  |  |  |  | X |  |  |
| Cucchi et al, 2023 |  |  |  |  |  |  | X |  |
| Lim et al, 2022 |  |  |  |  |  |  | X |  |
| D’Amato et al, 2015 |  |  |  |  |  |  | X |  |
| De Freitas et al, 2013 |  |  |  |  |  |  | X |  |
| Abaza et al, 2023 |  |  |  |  |  |  | X |  |
| Nahid et al, 2022 |  |  |  |  |  |  | X |  |
| Khaled et al, 2023 |  |  |  |  |  |  | X |  |
| Al Shaikh et al, 2020 |  |  |  |  |  |  | X |  |
| Marx et al, 2013 |  |  |  |  |  |  | X |  |
| Bahaa et al, 2023 |  |  |  |  |  |  | X |  |
| Li et al, 2023 |  |  |  |  |  |  |  | X |
