# Supplementary material for: Clinical Outcomes of Titanium Mesh for Alveolar Bone Augmentation: An Umbrella Review
Source: Clin Exp Dent Res. 2025 Dec 10;11(6):e70250. doi: 10.1002/cre2.70250 (PMC12690612; doi:10.1002/cre2.70250)
Supplement: Supplementary file 5 — Appendix 5: Grading the evidence. [file CRE2-11-e70250-s001.docx]

Appendix 5. Grading the evidence

| Study | Certainty | Downgrading due to |
| --- | --- | --- |
| Rasai-dal Polo et al 2014 | ⨁⨁◯◯, low | Risk of bias (-1), inconsistency (-1), AMSTAR 1-4 (-1) |
| Briguglio et al 2019 | ⨁◯◯◯, very low | Size (-1), risk of bias (-1), inconsistency (-1), AMSTAR 1-4 (-2) |
| Aceves-Argemi et al 2021 | ⨁⨁⨁◯, moderate | Inconsistency (-1), AMSTAR 1-4 (-1) |
| Abu-Mostafa et al 2022 | ⨁⨁◯◯, low | Size (-1), risk of bias (-1), inconsistency (-1), AMSTAR 1-4 (-1) |
| De Angelis et al 2023 | ⨁⨁◯◯, low | Size (-1), risk of bias (-1), inconsistency (-1), AMSTAR 1-4 (-1) |
| Anton et al 2024 | ⨁⨁◯◯, low | Size (-1), risk of bias (-1), inconsistency (-1) |
| Sabri et al 2024 | ⨁⨁⨁◯, moderate | Risk of bias (-1), inconsistency (-1) |
| Lorusso et al 2025 | ⨁⨁⨁◯, moderate | Size (-2) |

Note: AMSTAR questions 1-4 correspond to AMSTAR 2 questions 2, 4, 5, 6
